# Limited utility of optic nerve sheath diameter for monitoring intracranial pressure in patients with intracranial hemorrhage

**DOI:** 10.3389/fneur.2026.1932373

**Published:** 2026-10-08

**Authors:** Beschan Ahmad, Mario Maria Joachim Trappe, Lars-Olav Harnisch, Onnen Moerer, Ibrahim Allam, Fabian Kück, Ilko L. Maier

**Affiliations:** 1 Department of Neurology, University Medical Center Göttingen, Göttingen, Germany; 2 Department of Anesthesiology, University Medical Center Göttingen, Göttingen, Germany; 3 Department of Neurology, Saudi German Hospital, Dubai, United Arab Emirates; 4 Department of Medical Statistics, University Medical Center Göttingen, Göttingen, Germany

**Keywords:** intracranial hemorrhage, intracranial hypertension, intracranial pressure, neurocritical care, optic nerve sheath diameter

## Abstract

**Introduction:**

Monitoring intracranial pressure (ICP) is a key component in the management of patients with intracranial hemorrhage. Transorbital ultrasound (toUS)- based measurement of the optic nerve sheath diameter (ONSD) - has been proposed as a non-invasive surrogate for ICP assessment. However, substantial variability in measurement techniques and cut-off values has raised concerns regarding its reliability. This study aimed to evaluate the association between repeatedly measured ONSD and invasively measured ICP in patients with intracranial hemorrhage and to assess the ability of ONSD to reflect changes in ICP over time.

**Methods:**

In this prospective longitudinal study ONSD was measured daily using ultrasound at 3- and 6 mm posterior to the optic disc and compared with simultaneously recorded invasive ICP measurements. A total of 55 patients with intracranial hemorrhage were included, contributing repeated measurements over a mean of 5.47 ± 2.27 days.

**Results:**

Overall, the association between ICP and ONSD was weak. In the prespecified primary analysis, ICP was not associated with bilateral mean irONSD at 3 mm (*β* = 0.004 mm per mmHg, 95% CI − 0.003 to 0.011; *p* = 0.169; marginal R2 = 0.3%). At 6 mm, a small but statistically significant relationship was detectable at bilateral ICP mean values for outer-reflection ONSD [*β* = 0.010, 95% CI (0.002–0.019), *p* = 0.016; marginal r2 = 1.3%].

**Conclusion:**

ICP explains less than 2% of the variance in ONSD in this treated cohort with predominantly controlled ICP. These findings question its reliability as a stand-alone tool for serial monitoring of dynamic ICP changes in treated neurocritical care patients and therefore should not be interpreted as a direct substitute for invasive ICP or as a stand-alone measure of short-term ICP change.

## 1 Introduction

Intracranial pressure (ICP) monitoring is a cornerstone in the management of patients with intracranial hemorrhage, as elevated ICP is associated with poor outcomes including herniation and death (1). Invasive techniques such as intraventricular and intraparenchymal ICP measurement remain the gold standard to identify patients at risk. However, these methods are associated with relevant limitations including infection and hemorrhage, as well as logistical challenges particularly in emergency and resource limited settings (2). Consequently, there has been growing interest in non-invasive alternatives for ICP assessment.

Transorbital ultrasound (toUS)–based measurement of the optic nerve sheath diameter (ONSD) has emerged as a potential non-invasive surrogate marker for elevated ICP (3–5). The optic nerve sheath is continuous with the intracranial meninges and contains a perioptic subarachnoid space. Experimental infusion studies demonstrate that its diameter can change with cerebrospinal-fluid pressure, although the magnitude of this response varies among individuals (6, 7) Previous studies have reported varying degrees of correlation between ONSD and invasively measured ICP, with some suggesting diagnostic thresholds for elevated ICP (5, 8). However, clinical implications have been limited by heterogeneity in study designs, measurement protocols and proposed cutoff values. Notably, a systematic review demonstrated marked variability in reported normal ONSD values, ranging from 4.8 mm to 6.4 mm, depending on the measurement technique (5, 8). Moreover, patient related factors such as age, gender, and anatomical variability further contribute to measurement discrepancies (9). Furthermore, hemorrhagic entities differ in focal mass effect, hydrocephalus, CSF circulation, and intracranial compliance, a uniform relationship between ICP and perioptic CSF dynamics cannot be assumed.

Only a limited number of studies have evaluated whether within-patient changes in ONSD track contemporaneous changes in invasive ICP, and reported performance has varied across populations and pressure conditions (10–13). The reliability of ONSD for repeated short-interval monitoring therefore remains uncertain.

Against this background, recent methodological advances, including the introduction of standardized imaging protocols such as the CLOSED protocol, have sought to improve measurement consistency and interobserver reliability (9). Despite these efforts, the accuracy of toUS in capturing dynamic ICP changes and its clinical utility for Intracranial hemorrhage monitoring remains uncertain.

The aim of this study was to investigate the value of ONSD measurements for ICP monitoring in patients with intracranial hemorrhage. Specifically, we investigated the association between repeated ONSD measurements and invasively measured ICP, assessed the predictive value of ICP changes for alterations in the optic nerve (ON) sheath and compared measurements obtained at distinct sites using different techniques to identify the optimal ONSD-measurement approach. The prespecified primary endpoint was the association between simultaneously measured invasive ICP and bilateral mean irONSD measured 3 mm posterior to the optic disc. We hypothesized that higher ICP would be associated with a larger bilateral mean irONSD. Measurements at 6 mm, side-specific measurements, alternative anatomical definitions, longitudinal change analyses, inter-rater reliability and ROC analyses were secondary or exploratory.

## 2 Methods

### 2.1 Study design

This prospective longitudinal single-center observational cohort study was conducted in two neuro-intensive care units at one university medical center from May 2022 to December 2023, corresponding to a recruitment period of 19 months. Ethical approval (approval number: 28/2/22) was obtained from the institutional review board, and all procedures complied with established ethical standards. Written informed consent was obtained from all participants, or their legal representatives. Patients were enrolled consecutively to minimize selection bias.

### 2.2 Patient selection

Eligible patients were adults (age > 18 years) with acute intracranial hemorrhage requiring invasive ICP monitoring as part of routine neurocritical care. The cohort was designed to reflect a real-world neurocritical care population and therefore included different hemorrhagic entities, including spontaneous intracerebral hemorrhage (ICH) with or without intraventricular extension, subarachnoid hemorrhage, subdural hematoma, hemorrhagic transformation after ischemic stroke, and traumatic hemorrhagic lesions when orbital anatomy was unaffected. Patients with trauma affecting orbital anatomy or other contraindications to transorbital ultrasound were excluded. The decision to initiate invasive ICP monitoring was made by the treating neurocritical care team according to routine clinical practice and was based on the individual risk of intracranial hypertension, including factors such as impaired consciousness, hydrocephalus, intraventricular hemorrhage, mass effect, need for CSF diversion, or severe traumatic or spontaneous hemorrhagic brain injury. The study did not define independent indications for ICP probe or EVD placement. Furthermore, hydrocephalus, prior osmotherapy, CSF drainage, hematoma evacuation, and decompressive surgery were not prespecified exclusion criteria.

### 2.3 Measurement protocol

The prespecified primary endpoint was the association between simultaneously measured invasive intracranial pressure, and the bilateral mean inner-reflection ONSD (irONSD) at 3 mm posterior to the optic disc. We hypothesized that higher ICP would be associated with a larger bilateral mean ONSD. Measurements at 6 mm, side-specific measurements, alternative anatomical measurement definitions, longitudinal changes in ONSD and ICP, inter-rater reliability, and diagnostic performance were considered secondary exploratory outcomes.

Invasive ICP monitoring was performed using intraventricular catheters or parenchymal probes, considered the gold standard for direct ICP measurement. In parallel, toUS was performed daily for irONSD measurement at 3 mm posterior to the optic disc, considered as the clinically established reference measurement, for at least five consecutive days following ICP monitor placement. Given the high variability of measurement techniques and errors, additional measurements of the ON diameter, irONSD, outer-reflection ONSD (orONSD), and measurements at 6 mm at the same localizations were included as exploratory parameters to characterize whether alternative anatomical definitions or measurement depths might better capture ICP-related changes (Figure 1). There were exceptions for nine patients, which were measured in at least 8 days. Seven of the patients only had an interruption of one day. All diameters were recorded bilaterally at a standardized depth of 3 mm or 6 mm posterior to the optic disc parallel to the course of the ON, ensuring consistency across all readings. ONSD acquisition and ICP recording were performed simultaneously. The instantaneous ICP value displayed at the time of ultrasound acquisition was used as the reference measurement. Repeat examinations were performed after a mean interval of 22 ± 2 h, with a maximum deviation of 6 h from the planned 24-h interval.

**Figure 1:**
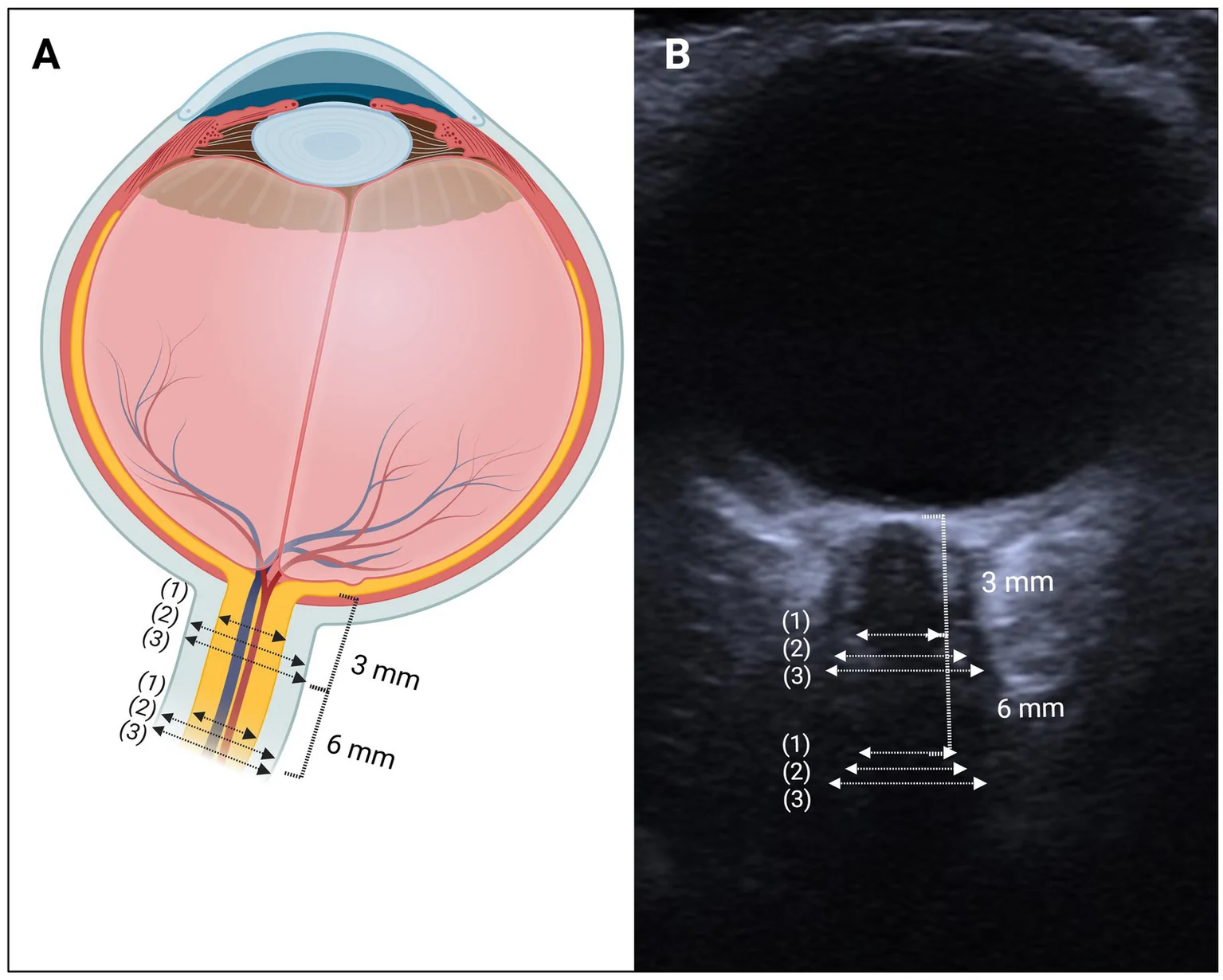
Optic nerve sheath and its surrounding subarachnoid space. Transorbital ultrasound was performed bilaterally through the closed eyelid in the supine 15° head-up position, using minimal probe pressure and three measurements per eye. ON, irONSD, and orONSD were measured between the inner nerve, inner dural, and outer dural reflections, respectively. **(A)** Schematic optic nerve and eye. **(B)** Transorbital ultrasound of the optic nerve and eye. (1) Optic nerve (ON) (2) Inner reflex optic nerve sheath diameter (3) Outer reflex optic nerve sheath diameter. All measurements were performed 3 mm and 6 mm behind the optic disc. Anatomical diagram was made with Biorender.

Additional patient-specific variables, including age, gender, partial pressure of carbon dioxide (paCO2), and positive end-expiratory pressure (PEEP), were meticulously recorded for each session to account for potential confounders. Changes in ICP (_Δ_ICP) over time were calculated to explore dynamic correlations with ONSD measurements.

ONSD measurements were acquired using a standardized local protocol. For the present report, acquisition and reporting elements were aligned with the quality domains in the 2024 international ONSD point-of-care ultrasonography checklist (8) using a Siemens ACUSON Juniper Ultrasound system (manufactured by Siemens, Germany, Berlin/Munich) with a linear probe and an adjusted preset for toUS including a mechanical index of 0.23 and thermal index < 1 (14). For potential technical issues General Electric Healthcare LOGIQ S8 ultrasound system (manufactured by GE Healthcare Technologies, Chicago, Illinois, USA) was used to maintain the settings for the measurements. ToUS was performed by a specialist with more than five years of experience certified by “Deutsche Gesellschaft für Ultraschall in der Medizin” (DEGUM e. V.). All toUS exams were reviewed and verified by a DEGUM-certified specialist with more than 10 years of toUS experience. In case of inconsistent findings or insufficient imaging quality, the toUS was repeated. Because ICP waveform verification and recalibration preceded ultrasound acquisition, the acquiring sonographer was not blinded to the current ICP. The second reader was not blinded to ICP values, clinical information, and the first reader’s measurements.

Optic nerve sheath diameter was assessed by transorbital ultrasound with the patient positioned supine and the upper body elevated at 15°. Measurements were performed bilaterally through the closed eyelid using a standardized axial insonation plane. For each patient, three consecutive measurements were obtained from the left eye and three consecutive measurements from the right eye. A generous layer of ultrasound gel was applied over the closed eyelid, and the probe was held with minimal pressure to avoid globe compression. ON was measured between inner reflections of the nerve, irONSD between the inner reflection of the *dura mater*, and orONSD between outer reflection of the *dura mater*. For each eye and definition, the three repeated measurements were summarized using the mean. Furthermore, ONSD was measured at two depths using three definitions (ON, irONSD, orONSD) and analyzed at three levels (mean of both eyes, ipsilateral, and contralateral). Images and video loops were stored locally.

A separate linear mixed-effects model was fitted for each combination. Each model had the respective ONSD measure (mm) as the outcome and ICP (mmHg) as fixed effect, with a random intercept per patient to account for the dependence between repeated measurements obtained from the same patient. A continuous-time first-order autoregressive correlation structure was specified for the within-patient residuals. The need for this structure was assessed by inspecting empirical variograms of the normalized residuals, which showed decreasing correlation with increasing time lag. Models were fitted by restricted maximum likelihood, which provides valid inference under a missing-at-random assumption. All available measurements were included in the analysis; no data was imputed. Negative ICP values were considered as missing.

### 2.4 Statistical analysis

The primary analysis evaluated the prespecified association between simultaneously measured invasive ICP and bilateral mean irONSD at 3 mm posterior to the optic disc. Bilateral mean ONSD was the dependent variable, and ICP, expressed per 1-mmHg increase, was the primary fixed effect in a longitudinal linear mixed-effects model. A positive regression coefficient was hypothesized, indicating an increase in ONSD with increasing ICP. A patient-specific random intercept and a continuous-time first-order autoregressive correlation structure were used to account for repeated measurements. All analyses involving measurements at 6 mm, individual eyes, alternative anatomical measurement definitions, or longitudinal changes were designated as secondary and exploratory.

Descriptive statistics were used to summarize patient demographics, baseline ICP, and ONSD measurements, reported as frequencies, mean ± standard deviation, and median (minimum, maximum, and interquartile range), as appropriate. Pearson’s correlation coefficients were calculated to assess relationships between ONSD, ICP, and demographic variables. Inter-rater reliability was evaluated using average-measure, absolute-agreement Intraclass Correlation Coefficients (ICC) for left, right, and mean bilateral values in a distinct population of 18 patients with acute intracranial hemorrhage treated on the same neuro-intensive care units. Moreover, the models were additionally fitted with age, sex, PEEP, and paCO₂ as further fixed effects, using the same random-effects and correlation structure.

Longitudinal changes were analyzed in a corresponding set of models, in which the change in ONSD from the preceding day (_Δ_ONSD, mm) was the outcome and the concurrent change in ICP (_Δ_ICP, mmHg) the fixed effect of interest. The preceding ICP value was included as a second fixed effect to account for the dependence of an observed change on the level from which it started. Random-effects and correlation structures were as above. A change in ICP greater than 5 mmHg was prespecified as relevant because of experience-based observations in clinical routine.

We report the estimated change in ONSD per 1 mmHg increase in ICP with 95% confidence intervals.

Linear regression was applied to day 0 measurements for cross sectional analysis. Receiver operating characteristic (ROC) analyses were performed to evaluate the predictive value of ONSD for elevated ICP and significant decreases in ICP, reporting ROC curve (AUC) with the 95% confidence interval [DeLong method (15) for single-day 0 data; Obuchowski method (16) for clustered data]. The significance level for all statistical tests was set at α = 5%, with no adjustment for multiple testing due to the exploratory nature of this study. For ROC analyses, elevated ICP was operationally defined as ICP > 20 mmHg. For exploratory analyses, an ICP decrease of more than 5 mmHg (_Δ_ICP < −5 mmHg) was used as a prespecified threshold based on clinical judgment.

Analyses were conducted in R (version 4.2.3; R Core Team, 2023), using nlme (version 3.1–162; Pinheiro, Bates, and R Core Team, 2023) for linear mixed-effects modeling, and the R package pROC (version 1.18.5) (17); as well as supplemental code for ROC analyses (18). ICCs were calculated using a two-way mixed effects intraclass correlation coefficient for absolute agreement based on the average of the included readers’ measurements [ICC(A,2)]. Patients/measurement cases were treated as random effects, whereas reader status was treated as a fixed effect because the readers were specifically selected and no generalization to other readers was intended. The unit of analysis was the corresponding ONSD measurement obtained from one patient at a defined examination time point, eye, measurement depth (3 or 6 mm), and measurement method with IBM SPSS statistics (v29.0.).

No formal *a priori* sample-size calculation was performed because the study was designed as an exploratory prospective cohort study. All consecutively eligible patients enrolled during the 19-month recruitment period were included.

## 3 Results

### 3.1 Patient characteristics

Of 301 ultrasound examination occasions acquired from 55 patients, 297 had an evaluable paired ICP value (Figure 2). Four occasions were excluded because of missing instantaneous ICP values. Analyses used all available paired observations; no values were imputed. On every day, ONSD was measured in more than 90% of the patients still under observation, whereas the number of patients under observation fell from 55 on day 0 to 30 on day 6 and 3 on day 9 (Supplementary Table 1). Mean age was 61.1 ± 16.2 (± SD) years, and 30 (54.5%) were male. The most frequent diagnoses were subarachnoid hemorrhage (SAH, 21.8%), ICH with intraventricular hemorrhage (IVH, 20%), subdural hematoma (14.5%), ICH without IVH (12.7%), and secondary hemorrhage after MCA infarction (12.7%).

**Figure 2:**
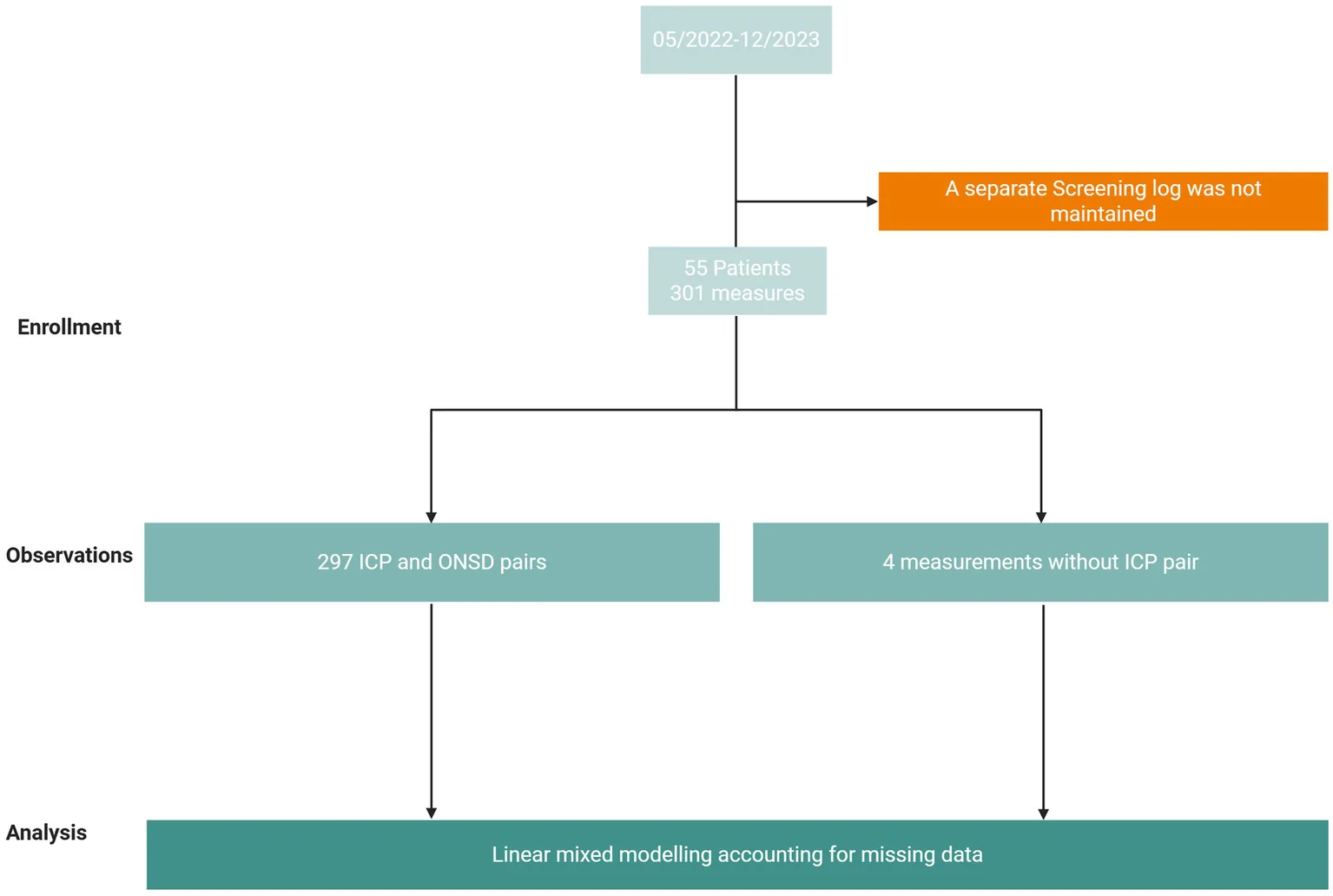
Participant and measurement flow. During the recruitment period, 55 consecutively enrolled patients contributed 301 ONSD measurement occasions. A separate screening log was not maintained. ICP data were missing for four occasions, leaving 297 paired ONSD–ICP observations for the linear mixed-effects analyses. All available paired observations were included without imputation.

Hydrocephalus, assessed by CT, was present in 30.9% of patients. Invasive ICP was measured using an intraparenchymal probe in 50 of 55 patients (90.9%) and via an EVD in 5 patients (9.1%). An EVD was present in 26 patients (47.3%), meaning that a substantial proportion of patients had an EVD in addition to intraparenchymal ICP monitoring. Mechanical ventilation was present in 50 patients (90.9%). (Table 1).

**Table 1:** Baseline characteristics of patients with intracranial hemorrhage and serial optic nerve sheath diameter (ONSD) measurements using transorbital ultrasound (*n* = 55).

Parameter	Value
Gender (Male, %)	30 (54.5)
Age (Mean years ± SD)	61.1 ± 16.2
Height (Mean cm ± SD)	173 ± 7
Weight (Mean kg ± SD)	80.5 ± 15.2
Localization of intracranial hemorrhage (*n*, %)	
Right hemisphere	28 (52.8)
Left hemisphere	17 (32.1)
Both hemispheres	5 (9.4)
Midline	2 (3.8)
Multilocular	1 (1.9)
Kind of intracranial hemorrhage (*n*, %)	
Subarachnoid hemorrhage	12 (21.8)
Typical intracerebral hemorrhage without intraventricular hemorrhage	7 (12.7)
Typical intracerebral hemorrhage with intraventricular hemorrhage	11 (20)
Atypical intracerebral hemorrhage	4 (7.3)
Subdural hematoma	8 (14.5)
Secondary hemorrhage after MCA infarction	7 (12.7)
Traumatic brain injury with intracerebral hemorrhage	6 (10.9)
Hydrocephalus (*n*, %)	
None	38 (69.1)
Malresorptive	1 (1.8)
Occlusional	6 (10.9)
Unclear	8 (14.5)
Mechanical ventilation (*n* yes, %)	50 (90.9)
ICP measurement via intraparenchymal probe (*n*, %)	
Right hemisphere	26 (47.3)
Left hemisphere	20 (36.4)
Both hemispheres	4 (7.3)
No probe	5 (9.1)
Extraventricular drainage (*n* yes, %)	26 (47.3)
Baseline PEEP (Mean cmH_2_O ± SD)	6.7 ± 1.6
Baseline paCO2 (Mean mmHg)	39.4 ± 4.9
Baseline SBP	127 ± 17
Baseline DBP	57.3 ± 11.6
Baseline MAP	80.2 ± 12.2

SD, standard deviation; MCA, middle cerebral artery; ICP, intracranial pressure; PEEP, positive endexpiratory pressure; SBP, systolic blood pressure; DBP, diastolic blood pressure; MAP, mean arterial pressure.

### 3.2 Baseline ICP and ONSD measurements

On day 0, median ICP was 10 mmHg (range 4–93). ON diameter measured 3.09 ± 0.27 mm (left) and 3.13 ± 0.29 mm (right). irONSD was 5.58 ± 0.59 mm (left) and 5.52 ± 0.57 mm (right), while orONSD measured 6.70 ± 0.68 mm (left) and 6.69 ± 0.69 mm (right). ICP and ON/ONSD values remained largely stable over time. Detailed ONSD and ICP measurements following probe or EVD insertion are provided in Supplementary Figure 1. Slightly higher mean ICP on day 0 compared with day 1 was driven by outliers without follow-up; from day 1 onward ICP remained stable (Supplementary Figure 1).

### 3.3 ONSD across ICP categories

Measurements were stratified into four ICP categories (<15, 15–20, 20–25, and ≥25 mmHg). Among 297 evaluable measurements, the majority were within the <15 mmHg group (*n* = 247), whereas only 7 measurements from 5 patients were ≥25 mmHg (Supplementary Table 2). Variability of ONSD measurements was greater at lower ICP levels. At ICP < 15 mmHg, SD for irONSD ranged from 0.56–0.62 mm and for orONSD from 0.63–0.69 mm, compared with 0.19–0.27 mm and 0.34–0.48 mm, respectively, at ICP ≥ 25 mmHg (Table 2).

**Table 2:** Optic nerve and optic nerve sheath diameter 3 and 6 mm behind the optic disc, dependent on the ICP.

Parameter		ICP level	<15	15–20	20–25	≥25	*p* value
		*n*	247	33	10	7	
3 mm	ON	Mean ± SD	3.11 ± 0.27	3.10 ± 0.26	3.22 ± 0.19	3.04 ± 0.12	0.93
	irONSD	Mean ± SD	5.56 ± 0.56	5.53 ± 0.55	5.66 ± 0.48	5.41 ± 0.19	0.89
	orONSD	Mean ± SD	6.71 ± 0.63	6.67 ± 0.58	6.79 ± 0.54	6.68 ± 0.48	0.22
6 mm	ON	Mean ± SD	3.45 ± 0.38	3.50 ± 0.33	3.58 ± 0.29	3.36 ± 0.17	0.58
	irONSD	Mean ± SD	6.07 ± 0.62	6.08 ± 0.58	6.17 ± 0.53	5.81 ± 0.27	0.82
	orONSD	Mean ± SD	7.17 ± 0.69	7.17 ± 0.60	7.25 ± 0.62	7.03 ± 0.34	0.75

ON, optic nerve; ICP, intracranial pressure; irONSD, inner reflex optic nerve sheath diameter; orONSD, outer reflex optic nerve sheath.

Across all six parameters (ON, irONSD, and orONSD at 3 and 6 mm), differences between ICP groups were small. ON diameters remained stable (3 mm: 3.04–3.22 mm, *p* = 0.93; 6 mm: 3.36–3.58 mm, *p* = 0.58). Similar stability was observed for irONSD (3 mm: 5.41–5.66 mm, *p* = 0.89; 6 mm: 5.81–6.17 mm, *p* = 0.82) and orONSD (3 mm: 6.67–6.79 mm, *p* = 0.22; 6 mm: 7.03–7.25 mm, *p* = 0.75). Although minor trends toward lower values in the ≥25 mmHg group were observed at 6 mm depth, no significant differences in ON or ONSD diameters were detected across ICP categories (Figure 3 and Table 2).

**Figure 3:**
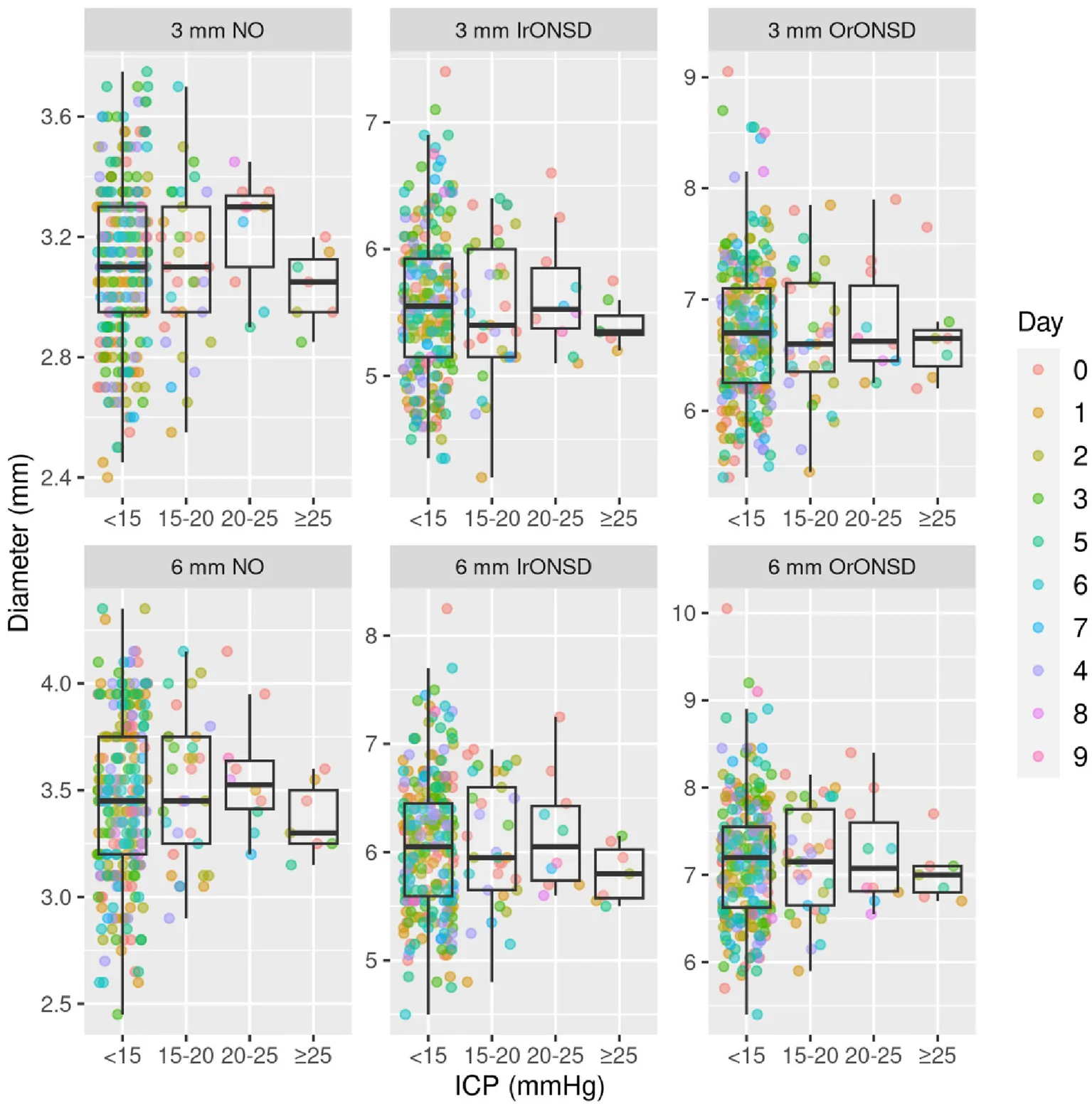
Scatterplots divided by the position of the measurement. Transorbital ultrasound was performed bilaterally through the closed eyelid in the supine 15° head-up position, using minimal probe pressure and three measurements per eye. ON, irONSD, and orONSD were measured between the inner nerve, inner dural, and outer dural reflections, respectively. Mean diameter of both sides. No significant differences were observed among the four intracranial pressure (ICP) groups across optic nerve diameter and optic nerve sheath diameter measurements, including inner and outer reflex diameters assessed at 3 mm and 6 mm posterior to the optic disc over the nine-day observation period. ON– optic nerve; ICP, intracranial pressure; irONSD, inner reflex optic nerve sheath diameter; orONSD, outer reflex optic nerve sheath.

### 3.4 Association between absolute ICP and ONSD

Linear mixed-effects models evaluated the influence of ICP on ON and ONSD at 3 mm and 6 mm posterior to the optic disc for ipsilateral, contralateral, and bilateral mean measurements. Age, sex, and PEEP showed no significant influence; paCO₂ had only a minimal effect on ON measurements.

In the prespecified primary analysis, higher ICP was not associated with a larger bilateral mean irONSD measured 3 mm posterior to the optic disc (*β* = 0.004 mm per mmHg, 95% CI − 0.003 to 0.0110; *p* = 0.169; marginal R^2^ approximately 0.3%). Thus, the prespecified directional hypothesis was not supported. All subsequent analyses of alternative measurement definitions, measurement depths, sides, and longitudinal changes were exploratory.

Ipsilateral to the pathology, ICP had no measurable effect at 3 mm on ONSD (*β* = −0.001, 95% CI − 0.006 to 0.005, *p* = 0.786; marginal R^2^ ≈ 0%), irONSD (*p* = 0.997), or orONSD (*p* = 0.598). At 6 mm ipsilateral, a borderline association was observed for orONSD (*β* = 0.013, 95% CI 0–0.026, *p* = 0.059; marginal R^2^ = 0.8%), whereas ON (*p* = 0.096) and irONSD (*p* = 0.145) remained non-significant.

Contralateral to the pathology, ICP effects at 3 mm were small and non-significant for ONSD (*β* = 0.003, *p* = 0.349) and irONSD (*β* = 0.006, *p* = 0.300); orONSD showed a near-significant trend (*β* = 0.009, *p* = 0.094). At 6 mm contralateral, ICP demonstrated a small but statistically significant association with ONSD (*β* = 0.009, 95% CI 0.001–0.017, *p* = 0.036; marginal R^2^ = 1.5%). A trend was present for irONSD (*β* = 0.012, *p* = 0.053), while orONSD remained non-significant (*p* = 0.089).

Across all measurement definitions and depths, the fitted associations were nearly flat relative to the observed scatter, with within-patient variation over the ICP range being small compared with differences between patients (Supplementary Figure 2).

For bilateral mean values, ICP showed no significant effect at 6 mm (*β* = 0.004, *p* = 0.134). irONSD demonstrated a borderline trend at 6 mm (*β* = 0.007, *p* = 0.060). In contrast, orONSD displayed small but statistically significant associations with ICP at both depths (3 mm: *β* = 0.010, 95% CI 0.003–0.016, *p* = 0.006; 6 mm: *β* = 0.010, 95% CI 0.002–0.019, *p* = 0.016). Across all models, explained variance remained low (marginal R^2^ ≤ 1.5%).

All associations between absolute ICP and ONSD can be seen in Table 3.

**Table 3:** Overview of varying influence of intracranial pressure across different positions and sides. Results of linear mixed-effects models with intracranial pressure as fixed effect. Results were similar in models additionally adjusted for age, sex, PEEP, and paCO₂.

Position	Variable	Side	*β* (ICP)	95% CI (ICP)	*p* (ICP)	Marginal r^2^ (%)
3 mm	ON	Ipsilateral	−0.001	[−0.006, 0.005]	0.786	0
		Contralateral	0.003	[−0.003, 0.01]	0.349	0.3
		Mean	0.001	[−0.006, 0.005]	0.699	0
	irONSD	Ipsilateral	0	[−0.01, 0.01]	0.997	0
		Contralateral	0.006	[−0.005, 0.016]	0.300	0.2
		Mean	0.004	[−0.003, 0.011]	0.169	0.3
	orONSD	Ipsilateral	0.003	[−0.008, 0.014]	0.598	0.1
		Contralateral	0.009	[−0.002, 0.019]	0.094	0.5
		Mean	0.010	[0.003, 0.016]	0.006*	1.3
6 mm	ON	Ipsilateral	0.007	[−0.001, 0.014]	0.096	0.7
		Contralateral	0.009	[0.001, 0.017]	0.036	1.5
		Mean	0.004	[−0.001, 0.009]	0.134	0.5
	irONSD	Ipsilateral	0.008	[−0.003, 0.019]	0.145	0.4
		Contralateral	0.012	[0, 0.024]	0.053	0.9
		Mean	0.007	[0, 0.014]	0.060	0.7
	orONSD	Ipsilateral	0.013	[0, 0.026]	0.059	0.8
		Contralateral	0.012	[−0.002, 0.026]	0.089	0.8
		Mean	0.010	[0.002, 0.019]	0.016	1.3

CI, confidence interval; ON, optic nerve; ICP, intracranial pressure; irONSD, inner reflex optic nerve sheath diameter; orONSD, outer reflex optic nerve sheath.

### 3.5 Longitudinal changes: _Δ_ICP and _Δ_ONSD

Longitudinal changes were stratified by _Δ_ICP (prespecified, <−5, −5 to 5, >5 mmHg). Most observations were within −5 to 5 mmHg (*n* = 179), whereas fewer measurements were observed in the _Δ_ICP < −5 (*n* = 21) and >5 (*n* = 23) groups (Figure 4). A consistent directional pattern was observed: decreases in ICP tended to coincide with decreases in ONSD and vice versa. However, most comparisons across _Δ_ICP categories did not reach statistical significance. Only irONSD at 6 mm showed a significant difference between _Δ_ICP groups (*p* = 0.019).

**Figure 4:**
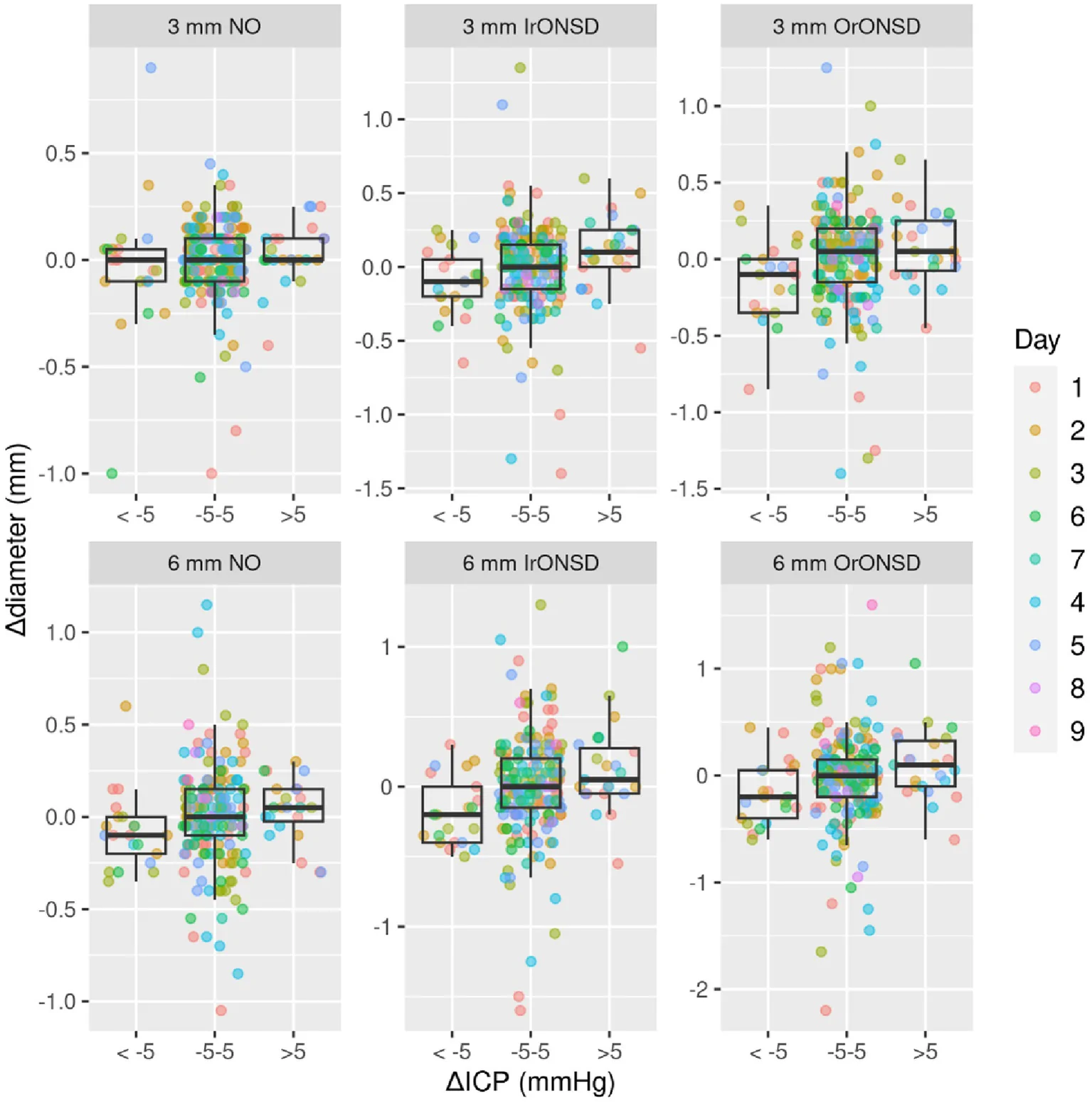
Scatterplots divided by days and dependent on _Δ_intracranial pressure (current minus previous). Transorbital ultrasound was performed bilaterally through the closed eyelid in the supine 15° head-up position, using minimal probe pressure and three measurements per eye. ON, irONSD, and orONSD were measured between the inner nerve, inner dural, and outer dural reflections, respectively. Values of contralateral side. Tendencies were observed among the three intracranial pressure groups in inner and outer optic nerve sheath diameter measurements (irONSD and orONSD), assessed at 3 mm and 6 mm posterior to the optic disc over the nine-day observation period; However, most comparisons were not statistically significant; irONSD at 6 mm differed nominally across delta-ICP categories (*p* = 0.019). This exploratory finding was not adjusted for multiplicity. ON, optic nerve; ICP, intracranial pressure; irONSD, inner reflex optic nerve sheath diameter; orONSD, outer reflex optic nerve sheath.

Mixed-effects models evaluating _Δ_ICP confirmed selective associations. For bilateral measurements, _Δ_ICP was significantly associated with _Δ_irONSD (3 mm: *β* = 0.010, 95% CI 0.000–0.019, *p* = 0.041; 6 mm: *β* = 0.017, 95% CI 0.006–0.029, *p* = 0.003) and _Δ_orONSD (3 mm: *β* = 0.010, 95% CI 0.000–0.020, *p* = 0.045; 6 mm: *β* = 0.017, 95% CI 0.003–0.031, *p* = 0.016), whereas ON showed no association. In ipsilateral-only analyses, no significant relationships were detected (all *p* > 0.15). Contralaterally, _Δ_ICP was associated with _Δ_irONSD (3 mm: *β* = 0.014, 95% CI 0.000–0.029, *p* = 0.048; 6 mm: *β* = 0.021, 95% CI 0.004–0.038, *p* = 0.014). Despite statistical significance, marginal R^2^ values remained low (≤ 4%), indicating limited explanatory power.

### 3.6 Inter-rater reliability

Inter-rater agreement was high. ICC for ON ranged from 0.76–0.87 at 3 mm and 0.79–0.85 at 6 mm. irONSD and orONSD showed excellent reproducibility (ICCs 0.92–0.95 and 0.93–0.95, respectively). All ICCs were significant (*p* < 0.001) (Supplementary Figure 3). Reliability analysis included 18 paired measurement cases from 18 randomly selected patients. The mean inter-reader difference was 0.03 mm. Limits of agreement were not estimated.

### 3.7 ROC analyses

ROC analyses assessed prediction of elevated ICP (>20 mmHg; 14 events in 11 of 55 patients across 297 occasions) and clinically relevant ICP decreases (_Δ_ICP < −5; 21 events in 19 of 50 patients across 232 occasions). Diagnostic performance was limited. On day 0, AUC values for orONSD were 0.714 (95% CI 0.488–0.941) at 3 mm and 0.652 (95% CI 0.420–0.884) at 6 mm, with sensitivity of 57% and specificity of 87–88% (3 mm) or sensitivity of 57% and specificity of 78% (6 mm); at optimal Youden cutoffs of 7.2 mm and 7.7 mm, respectively. Across all days, AUC values approached 0.5 (3 mm: 0.539; 95% CI 0.392–0.686; 6 mm: 0.482; 95% CI 0.334–0.631) with sensitivity of 100% and specificity of 18% (3 mm) or sensitivity of 93% and specificity of 27% (6 mm) at optimal Youden cutoffs of 6.2 mm and 6.7 mm, respectively. The best performance was observed for prediction of _Δ_ICP < −5 using irONSD at 6 mm (Figure 5).

**Figure 5:**
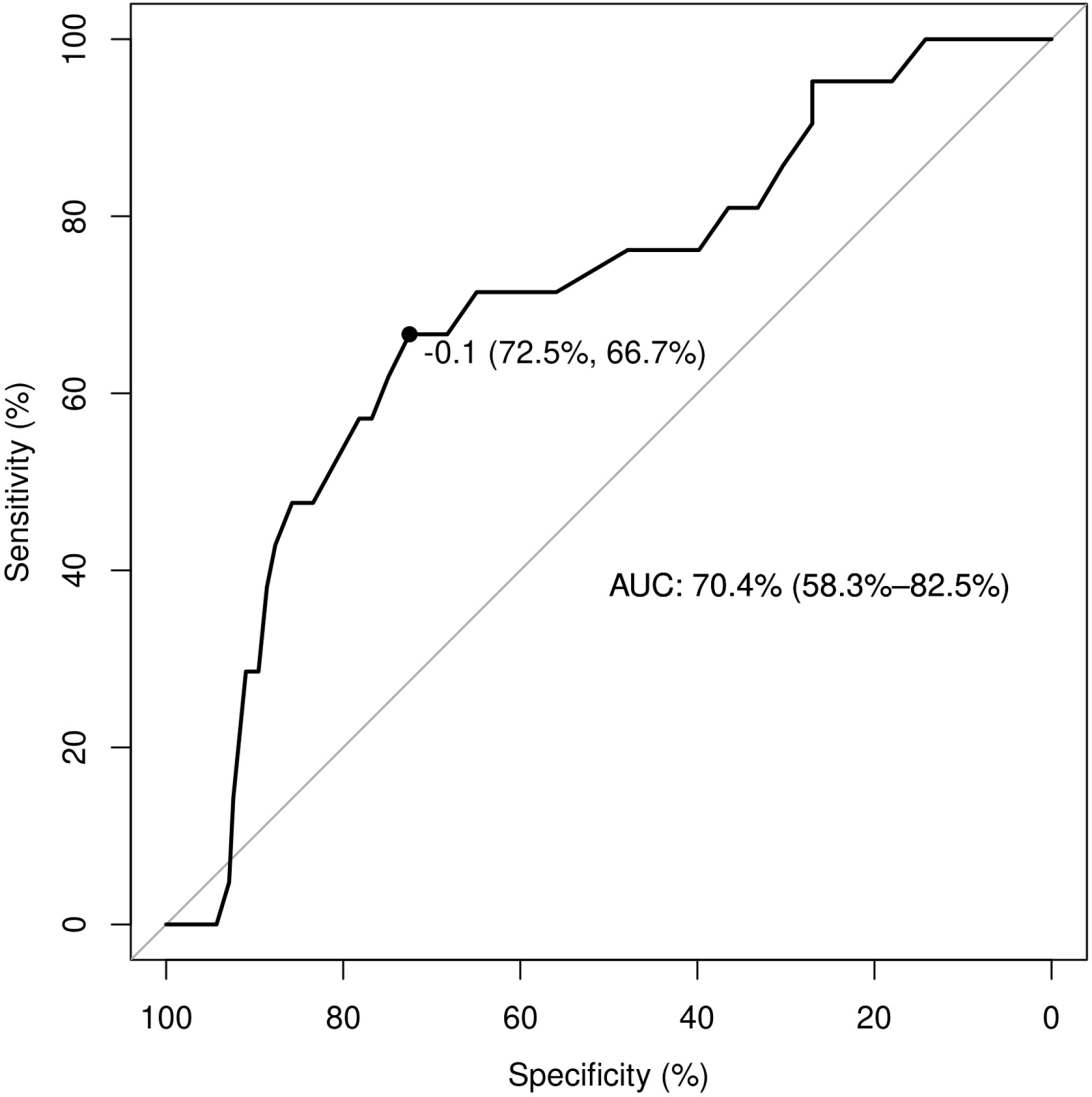
Receiver operating characteristic curve. Optimal cut-offs according to the Youden index (circle) and with sensitivity ≥ 90% (triangle), with corresponding specificity and sensitivity values, and the area under the curve (with the corresponding 95% confidence interval) for 6 mm behind the optic disc inner reflection optic sheath diameter and clinically relevant decrease in intracranial pressure (_Δ_ICP < −5). The mean of both sides was considered as representative value.

## 4 Discussion

In this study, we investigated determinants of ONSD variability using more than 300 individual measurements obtained from 55 patients across different treatment days, anatomical structures, and measurement positions posterior to the optic disc. By applying linear mixed-effects models, we specifically examined the contribution of ICP and changes in _Δ_ICP to both inter- and intraindividual ONSD variability. The principal finding of this study is that neither absolute ICP nor short-term changes in ICP explained a meaningful proportion of the observed variance in ONSD. When modeled independently, both parameters accounted for only a negligible fraction of ONSD variability. Although selected exploratory _Δ_ONSD measures showed nominal statistical associations with _Δ_ICP, the explained variance remained ≤4%, indicating no clinically meaningful relationship between short-term changes in ICP and ONSD.

The prespecified primary endpoint did not support the directional hypothesis that higher simultaneously measured invasive ICP would be associated with a larger bilateral mean ONSD measured 3 mm posterior to the optic disc. The estimated association was close to zero and explained virtually none of the observed variance. Although selected secondary exploratory measurements reached nominal statistical significance, these findings should not supersede the nonsignificant primary analysis and require cautious interpretation because of multiple testing and their very low explained variance.

The absence of a meaningful _Δ_ONSD– _Δ_ICP relationship raises important questions regarding the utility of toUS for detecting rapid ICP changes. Although ONSD has been proposed as a dynamic surrogate marker of ICP, our data suggests that this assumption may not hold true in the setting of acute intracranial hemorrhage, where ICP dynamics are complex and often rapidly evolving. Several anatomical and biomechanical factors likely contribute to the weak association between ONSD and ICP. Substantial interindividual variability in optic nerve sheath anatomy, differences in dural thickness, elasticity, and connective tissue composition, as well as the limited compliance of the sheath itself, have been described previously and may attenuate pressure transmission to the perioptic subarachnoid space (3, 7). These structural characteristics may render the optic nerve sheath relatively insensitive to transient pressure changes, particularly in acute pathological states. Notably, communication between optic nerve sheath and intracranial subarachnoid space, may lead to transmitted CSF pressure to the perioptic space. However, the magnitude and timing of this response could also be influenced by individual anatomy, intracranial compliance, chronicity of pressure elevation, CSF dynamics, lesion type, and treatment; ONSD should therefore not be expected to reproduce ICP on a one-to-one basis. However, these explanations are hypothesis-generating; anatomy, compliance, and pressure-transmission dynamics were not measured directly in this study. The temporal response of ONSD to changing ICP remains incompletely characterized. Experimental and clinical studies show that ONSD can change after manipulation of cerebrospinal-fluid pressure, but the magnitude, direction, and timing of this response vary across individuals and clinical settings (6, 10, 11, 19). Accordingly, brief or treated ICP changes may not be reproduced by ONSD on a one-to-one basis. This explanation remains hypothesis-generating because pressure-transmission dynamics were not measured directly in the present study.

Previous studies and meta-analyses have supported ONSD primarily as a screening marker for substantially elevated ICP, particularly in severe brain injury (4, 13, 20). By contrast, evidence for reliable within-patient tracking of short-term ICP changes is limited and inconsistent (10–12). Additionally, our study addressed a different clinical question: whether repeated ONSD measurements can track contemporaneous ICP and short-term ICP changes during ongoing neurocritical care. In our cohort, 247 of 297 evaluable measurements were obtained at ICP < 15 mmHg and only seven at ICP ≥ 25 mmHg. In addition, almost half of the patients had an EVD. Thus, our results should not be interpreted as refuting the established association between markedly elevated ICP and increased ONSD, but rather as questioning the utility of ONSD for serial monitoring in a treated population with predominantly controlled ICP.

Several limitations should be considered. The study population was heterogeneous and included different intracranial hemorrhage entities, including subarachnoid hemorrhage, ICH with and without intraventricular hemorrhage, subdural hematoma, and secondary hemorrhage after middle cerebral artery infarction. These entities differ in pathophysiology, CSF circulation, focal versus global pressure effects, and clinical indications for ICP monitoring. Although ipsilateral, contralateral, and bilateral ONSD measurements were analyzed to partly account for lesion laterality, this approach cannot fully address compartmental pressure gradients or disease-specific ICP dynamics. Due to the limited sample size, robust subgroup analyses according to hemorrhage subtype were not feasible.

Furthermore, the distribution of ICP values was imbalanced. Most measurements were obtained during normal or mildly elevated ICP, while only 7 of 297 evaluable measurements were recorded at ICP ≥ 25 mmHg. This restricted range limits the ability to assess ONSD performance during clinically relevant intracranial hypertension. Therefore, our findings should not be interpreted as excluding a potential role of ONSD for detecting sustained severe ICP elevation. Rather, they indicate that repeated ONSD measurements did not provide clinically meaningful information on ICP in a treated neurocritical care cohort with predominantly controlled ICP.

Additionally, ICP monitoring was performed according to clinical indication and not mandated by a standardized study protocol. Indications for monitoring may therefore have differed between hemorrhage subtypes. In addition, baseline ICP referred to the time point of study inclusion rather than hemorrhage onset or the first episode of pathological ICP elevation, and some patients had already received ICP-lowering interventions. These factors may have attenuated associations between simultaneous ICP and ONSD measurements. The specific ICP-lowering interventions prior to inclusion and their temporal relationship to individual ONSD measurements were not systematically recorded.

On another note, ICP measurement was not fully homogeneous. Most patients were monitored using intraparenchymal probes, whereas a minority underwent EVD-based monitoring. In approximately 10% of cases, ICP was measured contralateral to the side of interest or via EVD, which may not fully reflect local perioptic pressure dynamics, especially in focal lesions or compartmentalized pressure states.

Moreover, the ultrasound protocol included both established and exploratory measurements. ONSD at 3 mm posterior to the optic disc is the most widely used approach, whereas optic nerve diameter, inner-reflection ONSD, outer-reflection ONSD, and 6-mm measurements are less standardized. The inclusion of multiple parameters, depths, and side-specific analyses increases multiplicity and may generate statistically significant but clinically unstable findings. Accordingly, these analyses should be interpreted as exploratory. Importantly, even statistically significant associations explained only a very small proportion of ONSD variance.

Because multiple anatomical definitions, measurement depths, sides, and longitudinal comparisons were explored without adjustment for multiplicity, isolated statistically significant findings must be interpreted cautiously and may represent Type-I error. Accordingly, we consider these associations nominal and hypothesis-generating. Importantly, their marginal R^2^ values were very low, indicating that statistical significance did not translate into clinically meaningful explanatory or predictive value.

Furthermore, no formal *a priori* sample-size calculation was performed. Accordingly, the exploratory sample, particularly the small number of patients and observations with markedly elevated ICP, limits precision and does not exclude clinically relevant associations in a high-ICP population.

Finally, complete blinding was not feasible because ICP waveform verification and recalibration were required before ultrasound acquisition. Since transorbital ultrasound depends on manual image acquisition and caliper placement, observer bias cannot be excluded. Inter-rater reliability was assessed using only two experienced raters, which may overestimate reproducibility in routine clinical practice. Because the second reader was not blinded to the first reader’s measurements, the reported ICCs may overestimate agreement achievable with fully independent readings. Nevertheless, the high internal agreement supports the consistency of the ultrasound protocol within this study.

Despite these limitations, this study has several notable strengths. First, the analysis was based on a large number of repeated measurements obtained under real-world neurocritical care conditions, allowing for robust assessment of both inter- and intraindividual variability in ONSD. Second, the use of linear mixed-effects models enabled appropriate handling of repeated observations and confounders, improving the validity of variance attribution and reducing bias from unbalanced measurement schedules. Third, although only two raters were involved, the observed high intraclass correlation coefficient indicates strong internal measurement reliability, supporting the robustness of the ultrasound acquisition and measurement protocol within the study setting. Finally, the inclusion of multiple anatomical measurement positions posterior to the optic disc provides a more comprehensive characterization of ONSD variability than single-point approaches used in many prior studies.

Given these findings, reliance on isolated ONSD measurements for acute ICP monitoring appears unreliable in patients with ICH. ONSD may be more informative in clinical scenarios characterized by chronic or progressively elevated ICP, such as idiopathic intracranial hypertension or diffuse cerebral edema following resuscitation, where delayed but sustained ONSD expansion has been reported (21, 22). The performance of ONSD may differ across acute, chronic, focal, and global disorders of intracranial pressure; however, population-specific utility and temporal response require direct validation. Standardized protocols such as CLOSED have been proposed to improve acquisition consistency (9). Baseline anatomy and demographic characteristics may also contribute to between-patient variability (23, 24).

In this prospective longitudinal cohort of patients with intracranial hemorrhage requiring invasive ICP monitoring, repeated transorbital ultrasound measurements of ONSD showed no clinically meaningful association with absolute ICP or short-term ICP changes. Although selected exploratory measurements reached nominal statistical significance, the explained variance was very low and diagnostic performance was limited. Importantly, the cohort predominantly represented patients with controlled ICP under ongoing neurocritical care; therefore, these findings do not exclude a role for ONSD in detecting sustained severe intracranial hypertension. They instead question its reliability as a stand-alone tool for serial monitoring of dynamic ICP changes in treated neurocritical care patients, and therefore should not be interpreted as a direct substitute for invasive ICP or as a stand-alone measure of short-term ICP change (4, 5).

Further research should refine standardized acquisition, evaluate adjunctive sonographic parameters, and validate prespecified thresholds and endpoints in larger multicenter cohorts enriched for clinically relevant intracranial hypertension (22, 25).

## Data Availability

The raw data supporting the conclusions of this article will be made available by the authors, without undue reservation.

## Supplementary material

SUPPLEMENTARY FIGURE 1 Longitudinal course of transorbital ultrasound measurements and intracranial pressure. Transorbital ultrasound was performed bilaterally through the closed eyelid in the supine 15° head-up position, using minimal probe pressure and three measurements per eye. ON, irONSD, and orONSD were measured between the inner nerve, inner dural, and outer dural reflections, respectively. Daily mean diameters of the optic nerve (ON) and optic nerve sheath diameter measured using the inner dural reflex and outer dural reflex at 3 mm and 6 mm posterior to the optic disc are shown in the upper panel. The lower panel displays the corresponding daily mean intracranial pressure (ICP) values. Ultrasound-derived diameters remained largely stable over the observation period, whereas ICP showed greater day-to-day variability, with the highest value observed on day 0. ICP, intracranial pressure; irONSD, inner reflex optic nerve sheath diameter; orONSD, outer reflex optic nerve sheath.

SUPPLEMENTARY FIGURE 2 Transorbital ultrasound was performed bilaterally through the closed eyelid in the supine 15° head-up position, using minimal probe pressure and three measurements per eye. ON, irONSD, and orONSD were measured between the inner nerve, inner dural, and outer dural reflections, respectively. Clustered scatter plots of ONSD against ICP by measurement definition (columns) and depth (rows). Grey points are individual measurements, connected within patients; blue lines and bands show the fitted association from linear mixed-effects models with 95% confidence bands. β denotes the estimated change in diameter per 1 mmHg increase in ICP. One measurement with ICP 93 mmHg lies outside the plotted range but was included in all models.

SUPPLEMENTARY FIGURE 3 Transorbital ultrasound was performed bilaterally through the closed eyelid in the supine 15° head-up position, using minimal probe pressure and three measurements per eye. ON, irONSD, and orONSD were measured between the inner nerve, inner dural, and outer dural reflections, respectively. Inter-rater reliability of ultrasound-derived optic nerve measurements. Forest plot showing intraclass correlation coefficients (ICC) with 95% confidence intervals for measurements obtained by reader 1 and reader 2. Measurements are displayed separately for 3-mm and 6-mm assessment depths and stratified by side and measurement parameter. Vertical dashed reference lines indicate ICC thresholds of 0.75 and 0.90, commonly corresponding to good and excellent reliability, respectively. All ICC estimates were statistically significant (*p* < 0.001). L, left; R, right; LR, bilateral mean; ON, optic nerve diameter; irONSD, inner optic nerve sheath diameter; orONSD, outer optic nerve sheath diameter; ICC, intraclass correlation coefficient; CI, confidence interval.
