# Supplementary material for: Limited utility of optic nerve sheath diameter for monitoring intracranial pressure in patients with intracranial hemorrhage
Source: Front Neurol. 2026 Oct 8;17:1932373. doi: 10.3389/fneur.2026.1932373 (PMC13686862; doi:10.3389/fneur.2026.1932373)
Supplement: Supplementary file 1 [file Table_1.docx]

**Supplementary Table 1** Patients under observation and measurements performed per day. Days beyond the end of a patient’s observation period were not counted as missing. Percentages in the second column refer to the 55 patients observed on day 0; all other percentages refer to the patients still under observation on the respective day. The row "Patients under observation" totals person-days rather than patients. The same measurement occasions were available for all six ONSD measures (both positions and all three definitions), so the corresponding counts are identical.

| **Day** | **Patients under**  **observation, n (%)** | **ONSD measured**  **n (%)** | **ICP available**  **n** | **Complete ONSD–ICP**  **pairs, n (%)** | **Observation ended**  **on this day, n** |
| --- | --- | --- | --- | --- | --- |
| 0 | 55 (100) | 55 (100) | 53 | 53 (96) | 5 |
| 1 | 50 (91) | 47 (94) | 46 | 46 (92) | 4 |
| 2 | 46 (84) | 45 (98) | 44 | 44 (96) | 3 |
| 3 | 43 (78) | 41 (95) | 41 | 41 (95) | 3 |
| 4 | 40 (73) | 37 (92) | 37 | 37 (92) | 6 |
| 5 | 34 (62) | 31 (91) | 31 | 31 (91) | 4 |
| 6 | 30 (55) | 29 (97) | 29 | 29 (97) | 20 |
| 7 | 10 (18) | 10 (100) | 10 | 10 (100) | 7 |
| 8 | 3 (5) | 3 (100) | 3 | 3 (100) | 0 |
| 9 | 3 (5) | 3 (100) | 3 | 3 (100) | 3 |
| **Total** | **314** | **301 (96)** | **297** | **297 (95)** | **55** |

ONSD – optic nerve sheath diameter; ICP – intracranial pressure

**Supplementary Table 2**. Distribution of intracranial pressure measurements and unique patients across ICP categories. *Patients may contribute measurements to more than one ICP category; therefore, patient counts across categories are not mutually exclusive and do not sum to the total number of unique patients.

| **ICP (mmHg)** | **<15** | **15-20** | **20-25** | **≥25** | **total** |
| --- | --- | --- | --- | --- | --- |
| **Measurements** | 247 | 33 | 10 | 7 | 297 |
| **Patients** | 50 | 21 | 8 | 5 | 55* |

ICP – intracranial pressure
